# The Gender Effect of Health-Related Quality of Life in Hemodialysis Patients

**DOI:** 10.5812/numonthly.15934

**Published:** 2014-01-15

**Authors:** Eghlim Nemati, Mohsen Motalebi

**Affiliations:** 1 Nephrology and Urology Research Center, Baqiyatallah University of Medical Sciences, Tehran, IR Iran

**Keywords:** Quality of Life, Gender Identity, Renal Dialysis

***Dear Editor,***

We read with great interest the recent published outstanding article in your esteemed journal by Rostami et al. (1) entitled “Health-related quality of life in hemodialysis patients: an Iranian multi-center study”. The message of this valuable and huge study focused on drawing the attention to the biographic, socio-economic, and biochemical factors, which can influence on health-related quality of life (HRQoL) of hemodialysis (HD) patients.

The impact of gender on quality of life (QoL) has remained a challenge yet. Although Rostami and coworkers (1) showed that men had better QoL than women, other studies found that females had better (2) or equal (3) QoL when compared to males. There are some important subjects to note about gender effects on QoL which can help this matter: 1) higher prevalence and severity of psychological disorders such as major depressive disorder and anxiety disorder in females cause poorer HRQoL in women than men (4). 2) It seems females are more capable than males in providing and receiving emotional support. 3) Women benefit more from family support, but men receive more support from health care professionals (5). 4) Females with chronic kidney disease (CKD) have lower handgrip force, lower exercise tolerance, greater arm fat region, and less muscle area than males (6). 5) Higher prevalence of viral hepatitis in males on hemodialysis (7) can influence HRQoL in them. 6) It has been shown that other chronic disorders in HD patients can influence HRQoL (3). Anyway, it is important to note that there is a different for perception of QoL among various countries, areas, religious, ethnic groups, and races between both genders. It seems that a meta-analysis is needed to evaluate whether gender has any influence on QoL in HD patients.

Although Rostami et al. (1) showed no difference between QoL among HD patients with and without diabetes , Anees et al. (8) found the QOL of HD patients with diabetes was poor in comparison to patients without diabetes. In addition, they showed HD patients with non-diabetic causes of CKD had a better QOL in physical health scope than HD patients with diabetes (8). Patients with diabetes have multiorgan damage such as vision and cardiac problem, renal failure, cerebrovascular and peripheral vascular disease, which can lead to amputation and diminished physical health. Moreover, high rate of sleep disorders were demonstrated in patients with diabetes (9). Hence, it seems that HD patients who suffer from diabetes have worse QoL than others.

We agree that QoL in HD patients are poorer than normal population. It seems that besides preparing these patients for kidney transplantation and treatment of biochemical disorders, HD patients counseling can improve QoL. Abraham and coworkers (10) conducted a study to evaluate the effect of patients counseling on QoL and concluded patients counseling would improve QoL of HD patients in all domains; therefore, we can help these patients with our attention and counseling in addition to focus on treatment only.
